# The cellular specificity of haemopoietic stem cell proliferation regulators.

**DOI:** 10.1038/bjc.1984.180

**Published:** 1984-09

**Authors:** C. Tejero, N. G. Testa, B. I. Lord

## Abstract

The range of specificity of the CFU-S proliferation inhibitor and stimulator which are produced endogenously in the bone marrow has been investigated by measuring their effects on the proportion of cells killed by tritiated thymidine in mixed colony- (CFC-mix), erythroid burst- (BFU-E) and granulocyte/macrophage colony- (GM-CFC) forming cells as well as spleen colony forming units (CFU-S). Both CFU-S and CFC-mix were triggered by the stimulator into DNA-synthesis but BFU-E and GM-CFC were unaffected. The range of activity of the inhibitor was confined solely to the CFU-S population. This defined the specificity of both inhibitor and stimulator for the multipotent cells. The differential sensitivity of CFU-S and CFC-mix to the inhibitor and the lack of it for the stimulator suggested (a) that the CFC-mix is a relatively mature subpopulation of the CFU-S compartment and (b) that the relative sensitivity of a CFU-S to these factors changes as it matures from the early stem cell stage (Inhibitor-sensitive) to the more mature stages (Stimulator-sensitive) before becoming committed to a specific line of differentiation. The specificity of the inhibitor for haemopoietic stem cells suggests its potential value during chemotherapeutic procedures.


*Br. J. Cancer* (1984), **50**, 335–341

### The cellular specificity of haemopoietic stem cell proliferation regulators

C. Tejero[2], N.G. Testa[1] & B.I. Lord[1]

[1]*Paterson Laboratories, Christie Hospital & Holt Radium Institute, Withington, Manchester M20 9BX, UK, and* [2]*Departmento de Bioquimica, Facultad de Veterinaria, Universidad Complutense de Madrid, Madrid, Spain*

**Summary** The range of specificity of the CFU-S proliferation inhibitor and stimulator which are produced endogenously in the bone marrow has been investigated by measuring their effects on the proportion of cells killed by tritiated thymidine in mixed colony- (CFC-mix), erythroid burst- (BFU-E) and granulocyte/macrophage colony- (GM-CFC) forming cells as well as spleen colony forming units (CFU-S). Both CFU-S and CFC-mix were triggered by the stimulator into DNA-synthesis but BFU-E and GM-CFC were unaffected. The range of activity of the inhibitor was confined solely to the CFU-S population. This defined the specificity of both inhibitor and stimulator for the multipotent cells. The differential sensitivity of CFU-S and CFC-mix to the inhibitor and the lack of it for the stimulator suggested (a) that the CFC-mix is a relatively mature subpopulation of the CFU-S compartment and (b) that the relative sensitivity of a CFU-S to these factors changes as it matures from the early stem cell stage (Inhibitor-sensitive) to the more mature stages (Stimulator-sensitive) before becoming committed to a specific line of differentiation. The specificity of the inhibitor for haemopoietic stem cells suggests its potential value during chemotherapeutic procedures.

The proliferative status of haemopoietic stem cells measured as spleen colony-forming units or CFU-S (Till & McCulloch, 1961) can be regulated by endogenously derived inhibitory (Lord *et al.*, 1976; Frindel & Guigon, 1977; Cork *et al.*, 1981) and stimulatory (Frindel *et al.*, 1976; Lord *et al.*, 1977) factors. In 1972 Gidali & Lajtha, studying the kinetics of CFU-S behaviour following partial body irradiation came to the conclusion that the proliferation of CFU-S is regulated at a local level rather than by circulating factors. Retrospectively, the same conclusion was drawn from the work of Rencricca *et al.*, (1970) who demonstrated different levels of CFU-S proliferation in bone marrow and spleen. Since the factors we have described are obtained from fresh, viable marrow – the inhibitor from normal adult marrow in which CFU-S are proliferatively quiescent; the stimulator from post-irradiation marrow in which CFU-S are rapidly proliferating – we consider them probably to be at least a part of this local regulatory system. The inhibitor, described by Frindel & Guigon is obtained from other haemopoietic sources. It probably has a lower mol. wt and is interpreted by them to act humorally. The relationship between these two factors, if any, is, therefore, unclear and it should be pointed out that the data presented in this paper refer only to the inhibitor described by Lord *et al.*, (1976), and Cork *et al.*, (1981). Whether this is also true for the stimulatory factor is not known. Consequently, the properties described below cannot necessarily be extended to the factors described by Frindel and her colleagues. Nevertheless, like the inhibitor described by Frindel & Guigon (1977) our material is at least relatively specific to the CFU-S population in that it has no effect on GM-CFC (granulocyte/macrophage colony-forming cells, *in vitro*) (Lord *et al*, 1976). Similarly, Cork *et al.*, (1982) found that the stimulator would not trigger proliferatively suppressed GM-CFC back into cycle.

The *in vitro* colony techniques have now been expanded for assaying other early committed progenitor cells, *e.g.*, the erythroid burst-forming unit (BFU-E) and the mixed colony forming cell (CFC-mix). This latter cell type is particularly important since its growth *in vitro* produces a wide range of recognisable blood-cell lines and consequently it is considered to be a very close temporal relative of the pluripotent CFU-S population, if not the same cell. Its absolute relationship to the CFU-S, however, is not known. It has a sedimentation velocity similar to that of the CFU-S (Johnson, 1980) and because of its multipotency, it is almost certainly more primitive than the BFU-E. On the other hand, its self renewal capacity is less than that of the CFU-S (Metcalf *et al.*, 1979) though this could be an artefact of the *in vitro* technique. There is a tendency therefore to think of the CFC-mix as an intermediary between the CFU-S and the BFU-E. Measurements of the proliferative behaviour of CFC-mix, in parallel with the BFU-E, GM-CFC and CFU-S, have thus been used to examine more fully the specificity of the endogenously produced

Correspondence: B.I. Lord.
Received 7 March 1984; accepted 30 May 1984.

© The Macmillan Press Ltd., 1984

inhibitor and stimulator for CFU-S proliferation, to define the extent of their activities amongst the haemopoietic progenitor cells and to investigate further the temporal relationship of CFC-mix to CFU-S and BFU-E.

### Materials & methods

Bone marrow cells from male $BDF_1$ (C57Bl♀ x DBA2♂) mice aged 8 to 10 weeks or liver from DBA2 foetuses aged approximately 14 d were assayed for BFU-E, GM-CFC, CFC-mix and CFU-S. The proportion of cells in DNA-synthesis for each of these cell types was measured by the tritiated thymidine ($^3$HTdR) suicide technique (Becker *et al.*, 1965; Lord *et al.*, 1974). Inhibitor was obtained as a partially purified extract from normal bone marrow cells, NBME-IV (Lord *et al.*, 1976). Fresh murine bone marrow was suspended in saline at $\sim 10^7$ cells $ml^{-1}$ and maintained at room temperature for about 2 h before removing the cells by centrifugation. The cell free supernatant was then fractionated on Amicon Diaflo membranes and fraction IV, the 50-100 K dalton fraction, retained as the inhibitor. Stimulator (RBME-III) was similarly prepared as fraction III (the 30–50 K dalton Amicon fraction) from marrow regenerating after a dose of 4.5 Gy $\gamma$-rays (Lord *et al.*, 1977). The preparations were stored freeze dried until required.

### *BFU-E assay*

BFU-E colonies (bursts) were assayed by culturing $2 \times 10^5$ marrow cells from $BDF_1$ mice in $\alpha$-medium supplemented with 0.9% methylcellulose, 15% foetal calf serum, FCS (Gibco), 5% horse serum (Flow), 10% w/v bovine serum albumin, BSA (Sigma), 2 u $ml^{-1}$ erythropoietin (Connaught, Step III), $10^{-4}$ M $\alpha$-thioglycerol, $10^{-7}$ M sodium succinate and $10^{-3}$ M L-glutamine. Irradiated (16 Gy) bone marrow cells ($10^6$ $ml^{-1}$ of culture) were added as a source of burst-promoting activity, BPA. The cultures were incubated at 37°C in a fully humidified atmosphere of 5% $CO_2$ in air and the erythroid bursts were scored after 7–8 days under an inverted microscope at 50 or 78 x magnification. They were colonies composed solely of erythroid cells, recognised by their size, haemoglobinisation and characteristic arrangement in tight clusters of multicentric bursts.

### *GM-CFC assay*

GM-CFC colonies were assayed by culturing $10^5$ marrow cells from $BDF_1$ mice in 5 cm plastic petri dishes containing Fischer's medium with 25% horse serum and 0.3% agar. Heart cell conditioned medium was added (15%) as a source of colony stimulating activity, CSA. Triplicate cultures were incubated at 37°C in a humidified atmosphere containing 5% $CO_2$ in air. Colonies ($\geq 50$ cells) were counted after 7d of culture. They have the typical loose spherical cellular arrangement of granulocyte and/or macrophage colonies.

### *CFC-mix and BFU-E assays*

In later experiments, mixed colony cultures were established using Iscove's medium (Gibco Europe Ltd). Supplements to the medium were the same as for the BFU-E assay above except that FCS was increased to 20% and erythropoietin reduced to 1 u $ml^{-1}$. WEHI conditioned medium (10%) was used as a source of colony stimulating activity. Day 14 gestation foetal liver cells were plated at a final concentration of $10^5$ $ml^{-1}$, at 0.25 ml per well in triplicate wells (Costar) and incubated at 37°C in a fully humidified atmosphere containing 5% $CO_2$ and 5% $O_2$ in nitrogen. As these conditions also allowed optimum growth of BFU-E and GM-CFC, all three types of colony were scored in the same cultures at day 7. Mixed colonies always had an erythroid component as described for bursts above. In addition, macrophage and granulocytes in loose arrangements could be recognised within the colonies. Megakaryocytes recognisable by their size and refringence could also be found. On several occasions, single mixed colonies were picked from the cultures and their composition was confirmed by morphological examination of cytocentrifuge preparations.

### *CFU-S assay*

CFU-S were assayed by the spleen colony technique (Till & McCulloch, 1961). Recipient $BDF_1$ mice (10 per group) were irradiated (13.5 Gy, $^{60}$Co $\gamma$-rays at 0.85 Gy $h^{-1}$; Lord *et al.*, 1984) and injected i.v. with $5 \times 10^4$ foetal liver or normal bone marrow cells for the inhibitor or stimulator studies respectively. Mice were killed 9 days later, their spleens excised and fixed in Bouin's solution and the colonies counted.

### *Effects of inhibitor and stimulator on cell proliferation ($^3$HTdR suicide assay)*

*Stimulator* Normal bone marrow or foetal liver cells were incubated in paired tubes containing $5 \times 10^6$ cells in 1 ml Fischer's medium supplemented with 20% horse serum. Zero to 40 $\mu$g stimulator (RBME III) was added to each tube and the incubation continued for 2 h at 37°C. 7.4 MBq $^3$HTdR in 0.2 ml medium (Sp. Act. 555 GBq $mmol^{-1}$) was added to one tube of each pair and an equal volume of medium to the second tube for the last 30 min of incubation. The tubes were then quickly placed in ice to prevent further isotope incorporation. Finally, the cells were washed twice in medium containing cold thymidine (100 $\mu g\,ml^{-1}$) and assayed for their content of the various progenitor cell types.

*Inhibitor* Inhibitor (NBME-IV) was assayed in a similar manner to that for the stimulator. In this case, foetal liver cells were incubated for 2 h or 5 h. In either case, $^3$HTdR was added for the last 30 min of incubation only. The dose of NBME-IV used lay in the range of 0 to 80 $\mu g\,ml^{-1}$.

### Results

*Stimulator* The potential of the extract from regenerating marrow to stimulate (or trigger) proliferation in various classes of early haemopoietic progenitor cells was assessed in two series of experiments. Using a common suspension of normal adult bone marrow cells, CFU-S, BFU-E and GM-CFC were first tested independently. The results are shown in Table I. The RBME-III caused a small, though not dose-related, reduction in the plating efficiency of GM-CFC but not in that of BFU-E or on the seeding efficiency of CFU-S: the number of colonies produced remained unchanged over a wide dose range of RBME-III. The proportion of CFU-S in DNA-synthesis (*i.e.*, the proportion killed by $^3$HTdR) was increased from the non-significant level found in normal quiescent bone marrow CFU-S (6.5%) to ~30% ($P \ll 0.001$) with a dose of 10 $\mu g\,ml^{-1}$ or more. Although BFU-E and GM-CFC are rapidly proliferating in normal bone marrow their proliferation can be accelerated by appropriate stimuli. For example, in irradiated hypertransfused mice, the proportion of BFU-E in DNA-synthesis is increased from 30 to 63% (Iscove, 1977). Similarly, the proliferative index of GM-CFC was increased from 35% in normal bone marrow to 80% in regenerating bone marrow (Iscove *et al.*, 1970). However, the proliferation of BFU-E and GM-CFC was not stimulated by RBME-III doses up to 30 $\mu g\,ml^{-1}$. It should be noted that the marginal stimulation of GM-CFC with 20 $\mu g\,ml^{-1}$ was very small compared to that for CFU-S ($P=0.02$ compared with $P \ll 0.001$). However, there was no indication of a dose response (see below).

In the second series of experiments, foetal liver CFC-mix, BFU-E and GM-CFC were assayed in a single culture system. The results are shown in Table II. In this system, the plating efficiencies of all three cell types were unaffected by treatment

**Table I** Dose response of stimulator (RBME-III) on the proliferation of BFU-E, GM-CFC and CFU-S derived from normal $BDF_1$ bone marrow.

| | *Colonies $10^{-5}$ BM cells* $-^3HTdR$ | $+^3HTdR$ | *% in DNA-S (% killed by $^3HTdR$)* | $P^a$ |
|---|---|---|---|---|
| *CFU-S* | | | | |
| Control | 20.8±2.0 | 19.4±0.8 | 6.5±9.7 | |
| 5 μg RBME-III | 18.0±1.5 | 16.1±1.5 | 10.5±11.1 | 0.2 |
| 10 μg | 22.3±1.0 | 15.5±0.8 | 30.5±5.4 | ≪0.001 |
| 20 μg | 19.3±1.3 | 10.9±1.2 | 43.7±7.3 | ≪0.001 |
| 30 μg | 20.2±1.6 | 14.9±1.7 | 26.3±10.4 | <0.001 |
| *BFU-E* | | | | |
| Control | 14.8±2.3 | 9.2±1.3 | 37.9±13.1 | |
| 5 μg RBME-III | 13.3±3.0 | 7.6±1.6 | 42.5±17.8 | 0.42 |
| 10 μg | 14.8±1.9 | 9.3±0.9 | 37.1±10.0 | >0.9 |
| 20 μg | 19.3±2.7 | 12.0±0.3 | 37.9±8.8 | 0.85 |
| 30 μg | 14.2±1.2 | 8.7±0.7 | 38.8±7.1 | 0.75 |
| *GM-CFC* | | | | |
| Control | 212.6±19.8 | 161.8±8.7 | 23.9±8.2 | |
| 5 μg RBME-III | 161.0±10.1 | 110.9±6.2 | 31.1±5.8 | 0.19 |
| 10 μg | 159.5±9.9 | 112.3±12.5 | 29.6±9.0 | 0.28 |
| 20 μg | 163.0±7.6 | 102.0±10.4 | 37.4±7.0 | 0.023 |
| 30 μg | 165.8±9.8 | 125.9±8.9 | 24.1±7.0 | 0.82 |

Net 3–6 experiments at each dose.

Results shown ± standard error (results for % in S-phase are mean kills ± sd on the individual kills).

BM = normal bone marrow.

[a] $P$ = significance of difference from control using a two-tailed $\chi^2$ test.

**Table II** Dose response of stimulator (RBME-III) on the proliferation of CFC-mix, BFU-E and CFC derived from 14d DBA2 foetal liver

| | *Colonies* $10^{-5}$ *FL cells* $-{}^{3}HTdR$ | $+{}^{3}HTdR$ | *% in DNA-S (% killed by* $^{3}HTdR$*)* | $P^{a}$ |
|---|---|---|---|---|
| *CFC-mix* | | | | |
| Control | 14.8±1.3 | 11.0±0.9 | 25.7±8.9 | |
| 5 μg RBME-III | 16.0±4.5 | 5.6±1.5 | 65.0±13.6 | ≪0.001 |
| 10 μg | 19.2±3.5 | 8.4±2.3 | 56.3±14.4 | ≪0.001 |
| 20 μg | 14.3±2.7 | 6.3±1.7 | 56.0±14.5 | ≪0.001 |
| 30 μg | 18.4±1.8 | 7.2±1.0 | 60.9±6.6 | ≪0.001 |
| *BFU-E* | | | | |
| Control | 30.3±2.6 | 20.3±2.4 | 33.0±9.8 | |
| 5 μg RBME-III | 33.3±10.4 | 20.6±7.6 | 38.2±14.1 | 0.37 |
| 10 μg | 30.2±6.0 | 19.8±5.4 | 34.5±10.4 | 0.70 |
| 20 μg | 29.0±5.9 | 19.9±3.5 | 34.5±8.5 | 0.70 |
| 30 μg | 30.6±1.8 | 19.2±3.3 | 37.3±11.4 | 0.44 |
| *GM-CFC* | | | | |
| Control | 98.3±2.4 | 64.3±3.0 | 34.6±3.4 | |
| 5 μg RBME-III | 111.0±6.7 | 59.1±11.9 | 46.8±11.2 | 0.06 |
| 10 μg | 110.3±7.6 | 69.0±4.9 | 37.4±6.2 | 0.57 |
| 20 μg | 98.5±6.0 | 61.5±7.9 | 37.6±8.9 | >0.9 |
| 30 μg | 93.5±8.4 | 66.0±10.9 | 29.4±13.3 | 0.52 |

Net 3–6 experiments at each dose
Results shown ± standard error (results for % in S-phase are mean kills ± sd on the individual kills)
FL – foetal liver
$^{a}P$ – significance of difference from control using a two-tailed $\chi^2$ test.

with RBME-III. The $^{3}$HTdR suicide indices for BFU-E and GM-CFC were again unchanged ($P=0.06$ to $>0.9$). CFC-mix, however, were triggered; the proportion killed by $^{3}$HTdR increasing from 25.7% to the plateau level of ~60% with as little as 5 μg of the extract per ml ($P \ll 0.001$). The small stimulation of GM-CFC at 20 μg ml$^{-1}$ in the first series of experiments was not confirmed in these experiments where no change in proliferation was observed ($P>0.9$). Neither was there any reduction in plating efficiency in these experiments.

*Inhibitor* The inhibitor (NBME-IV) was assayed against foetal liver CFC-mix, BFU-E and GM-CFC in the combined culture technique and, simultaneously, against CFU-S. The results are shown in Table III. The plating and seeding efficiencies of all cell types were largely unaffected as was the $^{3}$HTdR suicide index of all but the CFU-S. In this latter case, 20 μg NBME-IV per ml of cells reduced the proportion killed by $^{3}$HTdR from 31.5% to 10.1% after 5 h incubation ($P \ll 0.001$). 40 μg ml$^{-1}$ were required for inhibition when the incubation period was 2 h only (36.4% to 9.7%) ($P \ll 0.001$). A marginal, but non-significant, reduction in kill was seen in GM-CFC after 5 h incubation at 40 μg ml$^{-1}$ ($P=0.14$).

In four of the fifteen sets of data for the *in vitro* colony forming cells there was an apparent stimulation of proliferation. These were, however, non-systematic changes and small by comparison with the highly significant inhibition of CFU-S proliferation.

### Discussion

The haemopoietic stem cell complex has recently been described as a population with a well defined age structure, the most primitive cells having a high self-renewal and a low differentiation capacity while the more mature cells have a lower self-renewal and increased differentiation capacity (Schofield, 1978; Rosendaal *et al.,* 1979). This population of spleen colony-forming units (CFU-S) gives rise to the more restricted progenitor cells which separately lead to the production of red blood cells (BFU-E), granulocytes or macrophages (GM-CFC) etc. The development of an *in vitro* assay system for pluripotent cells indicated a CFC-mix which is

**Table III** Dose response of inhibitor (NBME-IV) on the proliferation of BFU-E, GM-CFC & CFU-S derived from DBA2 foetal liver

| | *Colonies $10^{-5}$ FL cells* $-{}^{3}HTdR$ | $+{}^{3}HTdR$ | *% in DNA synthesis (% killed by ${}^{3}HTdR$)* | $P^{a}$ |
|---|---|---|---|---|
| *2 hrs CFC-mix* | | | | |
| Control | 14.3±3.6 | 9.8±4.2 | 31.3±6.4 | |
| 20 μg NBME-IV | 12.0±5.7 | 6.8±3.9 | 43.2±4.4 | 0.06 |
| 40 μg | 11.0±3.5 | 7.0±0.9 | 36.3±9.4 | 0.37 |
| 80 μg | 13.0±4.4 | 9.4±2.5 | 28.0±3.0 | 0.72 |
| *5 hrs* | | | | |
| Control | 10.0±4.6 | 5.6±2.1 | 44.0±23.5 | |
| 20 | 9.3±1.5 | 4.5±3.0 | 51.7±18.3 | 0.21 |
| 40 | 10.7±1.5 | 3.8±1.5 | 64.5±12.1 | >0.002 |
| *2 hrs BFU-E* | | | | |
| Control | 28.3±6.4 | 16.1±4.4 | 42.3±4.7 | |
| 20 μg | 20.7±8.4 | 7.6±3.4 | 63.3±3.9 | 0.002 |
| 40 μg | 24.0±9.2 | 10.1±1.9 | 58.0±4.9 | 0.017 |
| 80 μg | 25.7±9.6 | 13.4±5.1 | 48.0±5.3 | 0.32 |
| *5 hrs* | | | | |
| Control | 24.7±9.8 | 15.8±5.6 | 36.0±7.9 | |
| 20 | 20.0±6.4 | 15.3±4.0 | 23.5±1.5 | 0.076 |
| 40 | 16.3±3.5 | 7.7±2.2 | 52.7±1.5 | 0.012 |
| *2 hrs GM-CFC* | | | | |
| Control | 71.0±22.2 | 48.1±13.4 | 32.3±6.8 | |
| 20 | 55.0±12.5 | 34.5±7.9 | 37.3±6.1 | 0.37 |
| 40 | 60.7±14.8 | 39.2±10.5 | 35.5±9.8 | 0.52 |
| 80 | 68.3±14.3 | 47.9±13.0 | 29.8±7.8 | 0.80 |
| *5 hrs* | | | | |
| Control | 43.0±16.6 | 25.2±11.0 | 41.3±4.5 | |
| 20 | 40.0±10.9 | 21.5±4.6 | 46.3±8.8 | 0.39 |
| 40 | 45.3±15.2 | 35.0±7.0 | 29.8±7.3 | 0.12 |
| *2 hrs CFU-S* | | | | |
| Control | 11.8±1.1 | 7.5±0.8 | 36.4±6.9 | |
| 20 | 12.1±1.2 | 9.0±0.5 | 25.8±4.2 | 0.14 |
| 40 | 10.3±0.6 | 9.3±1.2 | 9.7±4.5 | ≪0.001 |
| 80 | 11.2±0.9 | 10.3±0.7 | 8.0±5.9 | ≪0.001 |
| *5 hrs* | | | | |
| Control | 14.5±2.7 | 9.9±0.4 | 31.5±2.4 | |
| 20 | 12.7±1.7 | 11.4±0.9 | 10.1±7.3 | ≪0.001 |
| 40 | 12.2±1.9 | 11.9±1.3 | 1.0±6.4 | ≪0.001 |

Net 3–6 experiments at each dose
Results shown ± se (see **Tables I & II**).
FL – foetal liver
2 h & 5 h represents the duration of incubation of the assay cells with the inhibitor, NBME-IV.
[a]*P* – significance of difference from control using a two-tailed $\chi^2$ test.

pluripotent, has a degree of self renewal capacity and therefore, is at least very close to the CFU-S.

RBME-III has been shown to stimulate normal CFU-S but not GM-CFC whose proliferative activity has been suppressed (Cork *et al.,* 1982). NBME-IV, on the other hand, inhibits regenerating CFU-S but not proliferating GM-CFC (Lord *et al.,* 1976). These two factors have, therefore, been taken as proliferation regulators which are active specifically against the CFU-S population. These observations have now been extended to include the other major derivative of the CFU-S, the erythroid progenitor cells. By including also the CFC-mix, the extent of the specificity could be better defined.

These experiments confirmed the specificity of both NBME-IV and RBME-III for the pluripotent cell forms. The stimulator, however, was active against both the CFU-S and the CFC-mix (Tables I and II) while the inhibitor was active against the CFU-S only (Table III). These observations are in accord with a separate series of experiments (Wright & Lord, in preparation) where it was found that the more primitive (12d) CFU-S are relatively more responsive to inhibitor while the maturer (7d) CFU-S are relatively more responsive to stimulator. Thus, the current observation that the stimulator's range of activity extends to the CFC-mix further suggests that this population arises at the more mature end of the CFU-S population. The range of activity of the inhibitor is probably restricted specifically to the purely CFU-S compartment. Once definable as a precursor committed to a specific line of development, however, a cell is no longer responsive to either of these stem cell regulating factors. Figure 1 illustrates diagramatically the temporal relationships between the stem and committed cell populations and their relative sensitivities to inhibitor and stimulator. It is interesting to note that GM-CFC demonstrate a similar heterogeneity in response to simulation in that a cell's sensitivity to colony stimulating activity appears to be related to its clone size potential (Francis *et al.,* 1981).

**Figure 1** Diagram illustrating the changing sensitivity of a CFU-S to inhibitor and stimulator as it progresses from the early, primitive stem cell stage through to the more mature CFC-mix and committed precursor cell stages.

The ability to protect haemopoietic stem cells against the cytotoxic effects of S-phase specific agents such as is illustrated by the specificity of this inhibitor means that extra flexibility in the use of such drugs for chemotherapy should be possible. Preliminary experiments have illustrated its effectiveness *in vivo* (Lord and Wright, 1982) in a similar manner to that of the inhibitor described by Frindel and Guigon (1977).

This work was supported by grants from the Cancer Research Campaign. We would like to thank Mrs L.B. Woolford for expert technical assistance.

### References

BECKER, A.J., McCULLOCH, E.A., SIMINOVITCH, L. & TILL, J.E. (1965). The effect of differing demands for blood cell production on DNA synthesis by hemopoietic colony forming cells of mice. *Blood,* **26,** 296.

CORK, M.J., ANDERSON, I., THOMAS, D.B. & RICHES, A.C. (1981). Regulation of the growth fraction of CFU-S by an inhibitor produced by bone marrow. *Leukaemia Res.,* **5,** 101.

CORK, M.J., WRIGHT, E.G. & RICHES, A.C. (1982). Regulation of murine granulocyte-macrophage progenitor cell and haemopoietic stem cell proliferation by factors produced in human foetal liver. *Leuk. Res.,* **6,** 553.

FRANCIS, G.E., BERNEY, J.J., BODGER, M.P., BOL, S.J.L., WING, M.A. & HOFFBRAND, A.V. (1981). Clone size potential and sensitivity to colony-stimulating activity: differentiation linked properties of granulocyte-macrophage progenitor cells. *Stem Cells,* **1,** 124.

FRINDEL, E., CROIZAT, H. & VASSORT, F. (1976). Stimulating factors liberated by treated bone marrow: in vitro effect on CFU-kinetics. *Exp. Hematol,* **4,** 56.

FRINDEL, E. & GUIGON, M. (1977). Inhibition of CFU entry into cycle by a bone marrow extract. *Exp. Hematol,* **5,** 74.

GIDALI, J. & LAJTHA, L.G. (1972). Regulation of haemopoietic stem cell turnover in partially irradiated mice. *Cell Tissue Kinet.,* **5,** 147.

ISCOVE, N.N. (1977). The role of erythropoietin in regulation of population size and cell cycling of early and late erythroid precursors in mouse bone marrow. *Cell Tissue Kinet.,* **10,** 323.

ISCOVE, N.N., TILL, J.E. & McCULLOCH, E.A. (1970). The proliferative states of mouse granulopoietic progenitor cells. *Proc. Soc. Exp. Biol. Med.,* **13,** 33.

JOHNSON, G.R. (1980). Colony formation in agar by adult bone marrow multipotential hemopoietic cells. *J. Cell Physiol.,* **103,** 371.

LORD, B.I., HENDRY, J.H., KEENE, J.P., HODGSON, B.W., XU- C-X., REZVANI, M. & JORDAN, T.J. (1984). A comparison of low and high dose-rate radiation for recipient mice in spleen-colony studies. *Cell Tissue Kinet.*, **17**, 323.

LORD, B.I., LAJTHA, L.G. & GIDALI, J. (1974). Measurement of the kinetic status of bone marrow precursor cells: three cautionary tales. *Cell Tissue Kinet.*, **7**, 507.

LORD, B.I., MORI, K.J. & WRIGHT, E.G. (1977). A stimulator of stem cell proliferation in regenerating bone marrow. *Biomed. Exp.*, **27**, 223.

LORD, B.I., MORI, K.J., WRIGHT, E.G. & LAJTHA, L.G. (1976). An inhibitor of stem cell proliferation in normal bone marrow. *Br. J. Haematol.*, **34**, 441.

LORD, B.I. & WRIGHT, E.G. (1982). Potential therapeutic value of endogenous stem cell proliferation regulators. *In* Progress in Cancer Research and Therapy: Maturation Factors and Cancer (ed. M.A.S. Moore) **23**, 323.

METCALF, D., JOHNSON, G.R. & MANDEL, T.E. (1979). Colony formation in agar by multi-potential hemopoietic cells. *J. Cell Physiol.*, **98**, 401.

RENCRICCA, N.J., RIZZOLI, V., HOWARD, D., DUFFY, P. & STOHLMAN, F. Jr. (1970). Stem cell migration and proliferation during severe anaemia. *Blood*, **36**, 764.

ROSENDAAL, M., HODGSON, G.S. & BRADLEY, T.R. (1979). Organization of haemopoietic stem cells: the generation-age hypothesis. *Cell Tissue Kinet.*, **12**, 17.

SCHOFIELD, R., (1978). The relationship between the spleen colony-forming cell and the haemopoietic stem cell: a hypothesis. *Blood Cells*, **4**, 7.

TILL, J.E. & McCULLOCH, E.A. (1961). A direct measurement of the radiation sensitivity of normal mouse bone marrow cells. *Radiat. Res.*, **14**, 213.


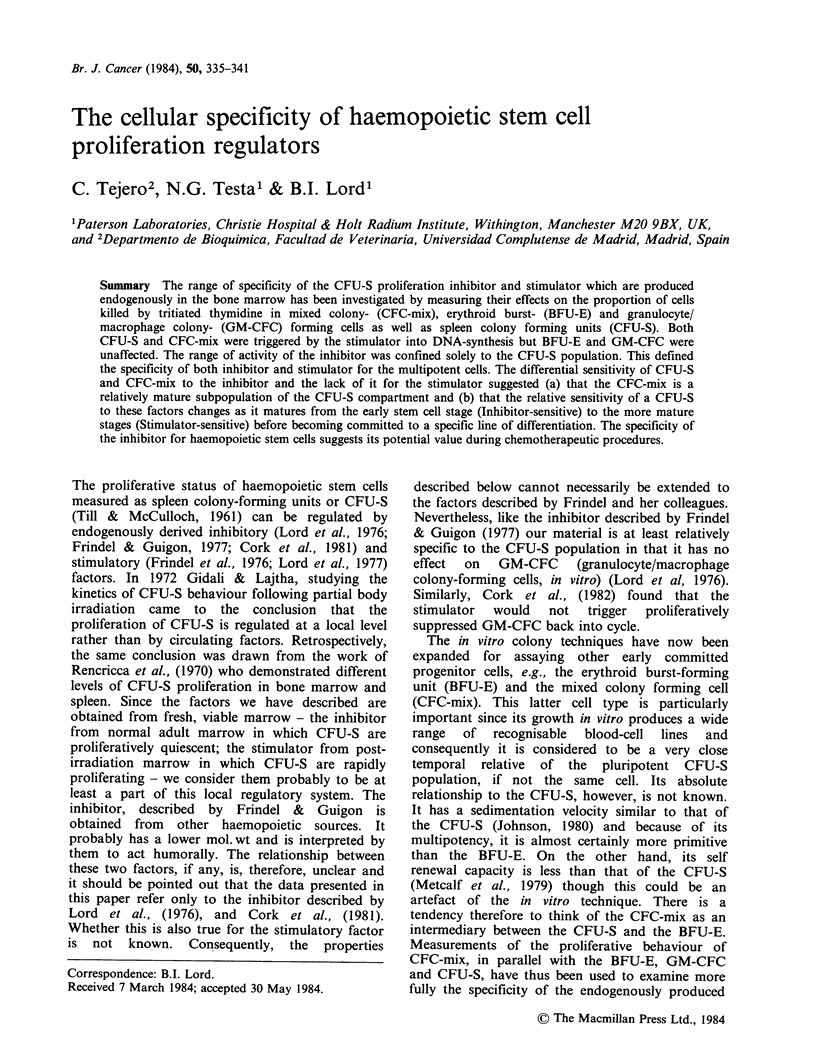

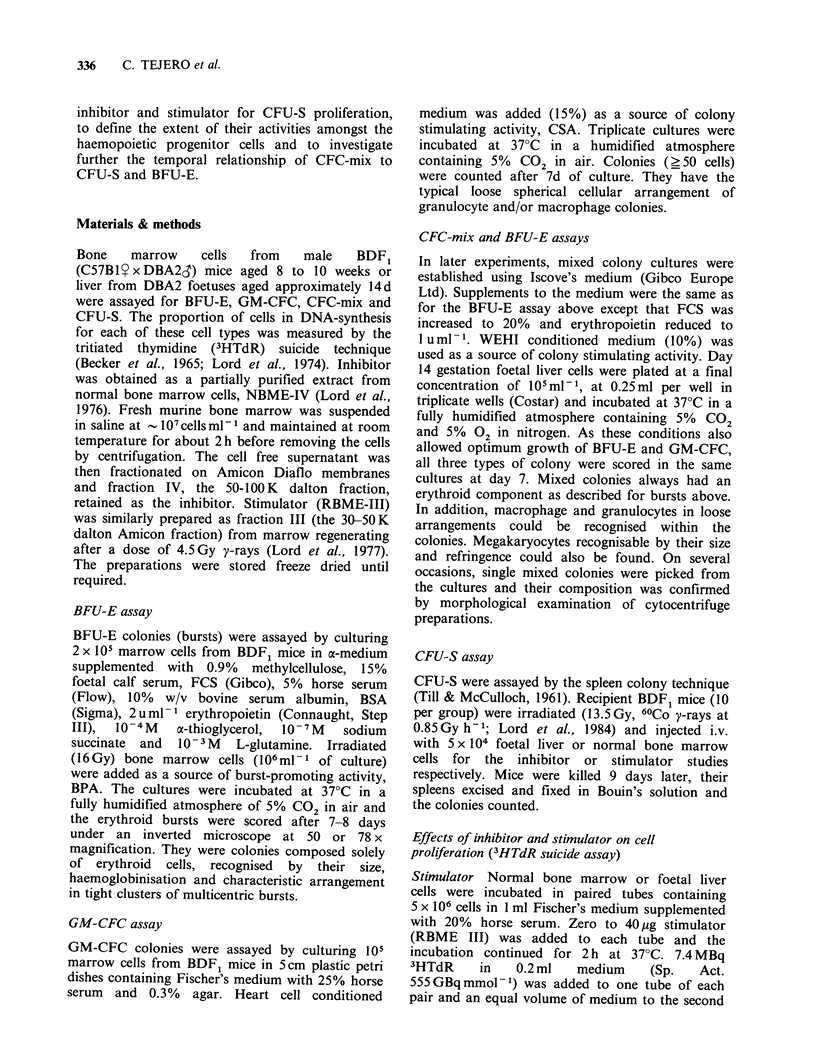

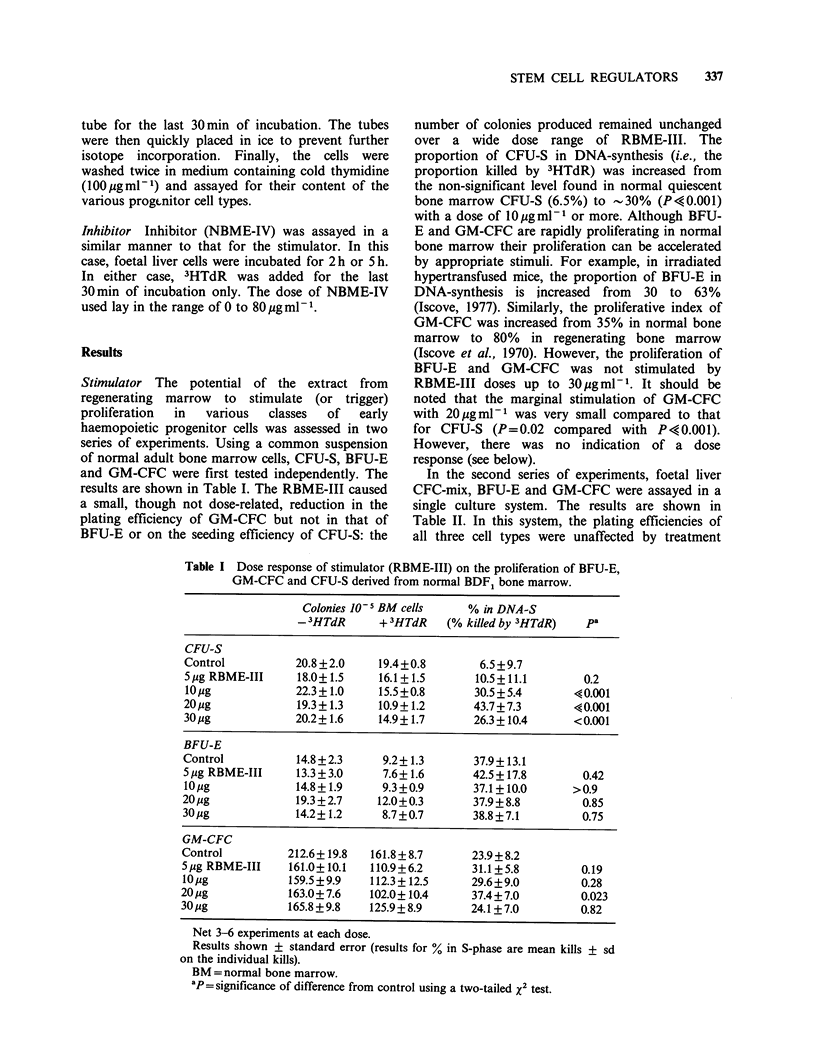

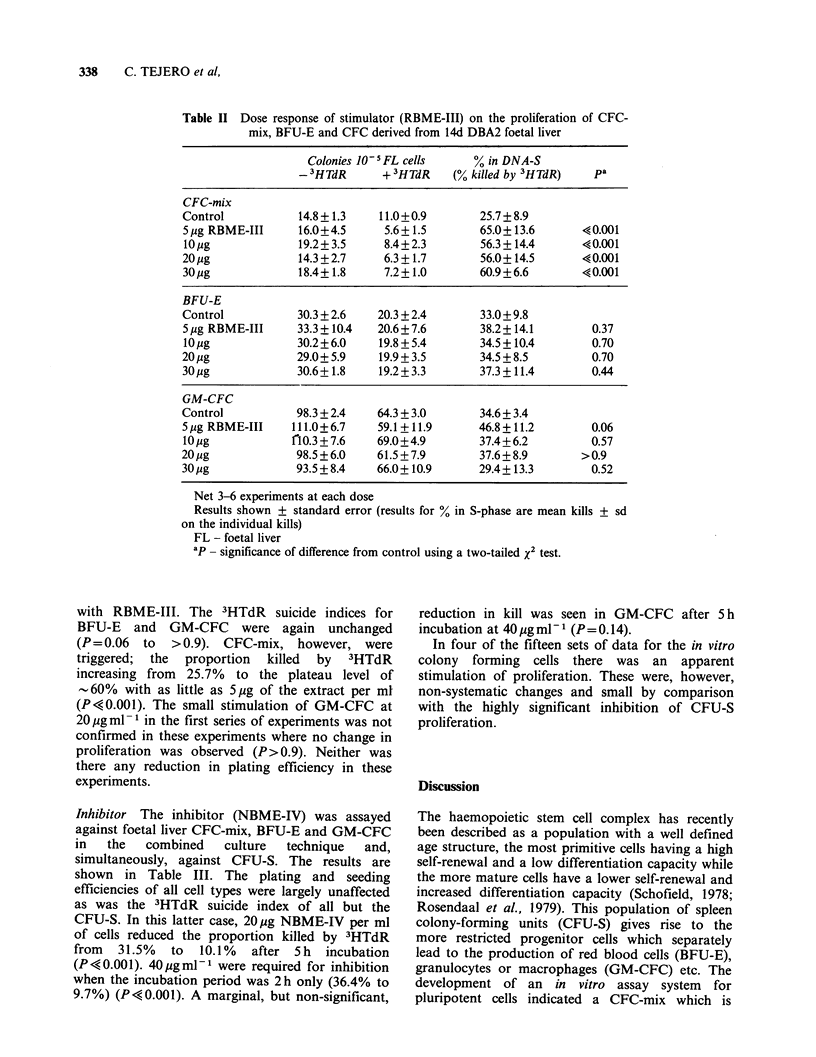

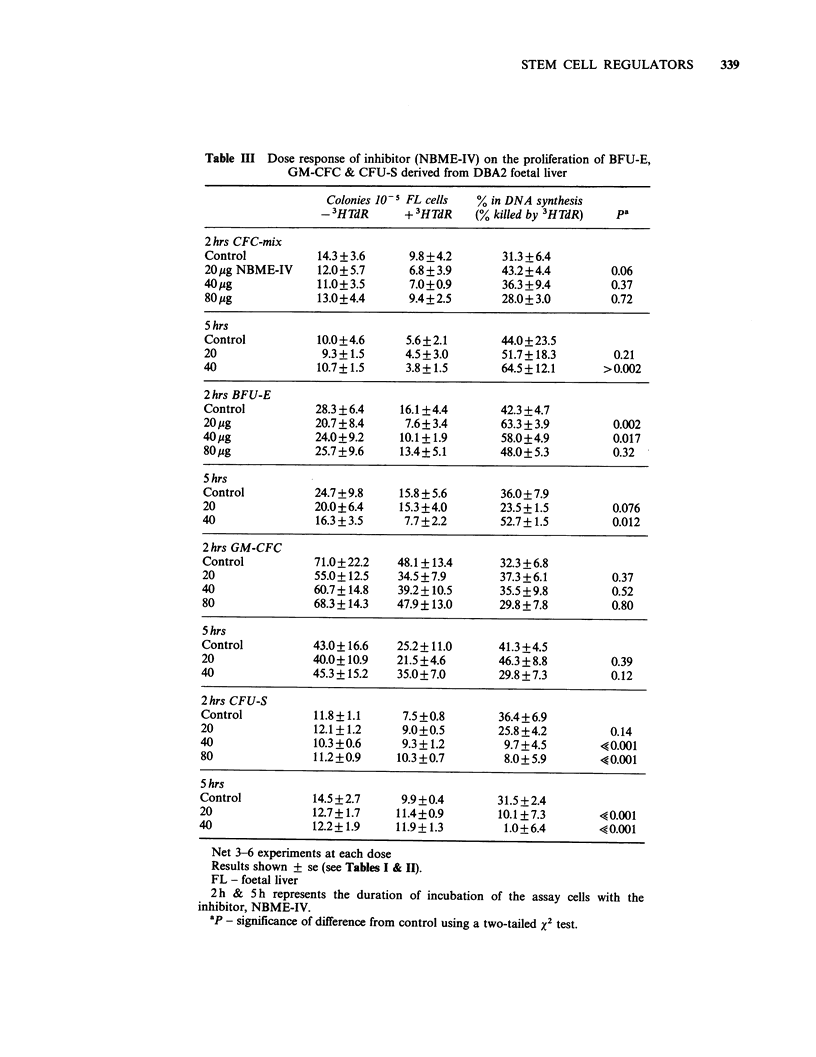

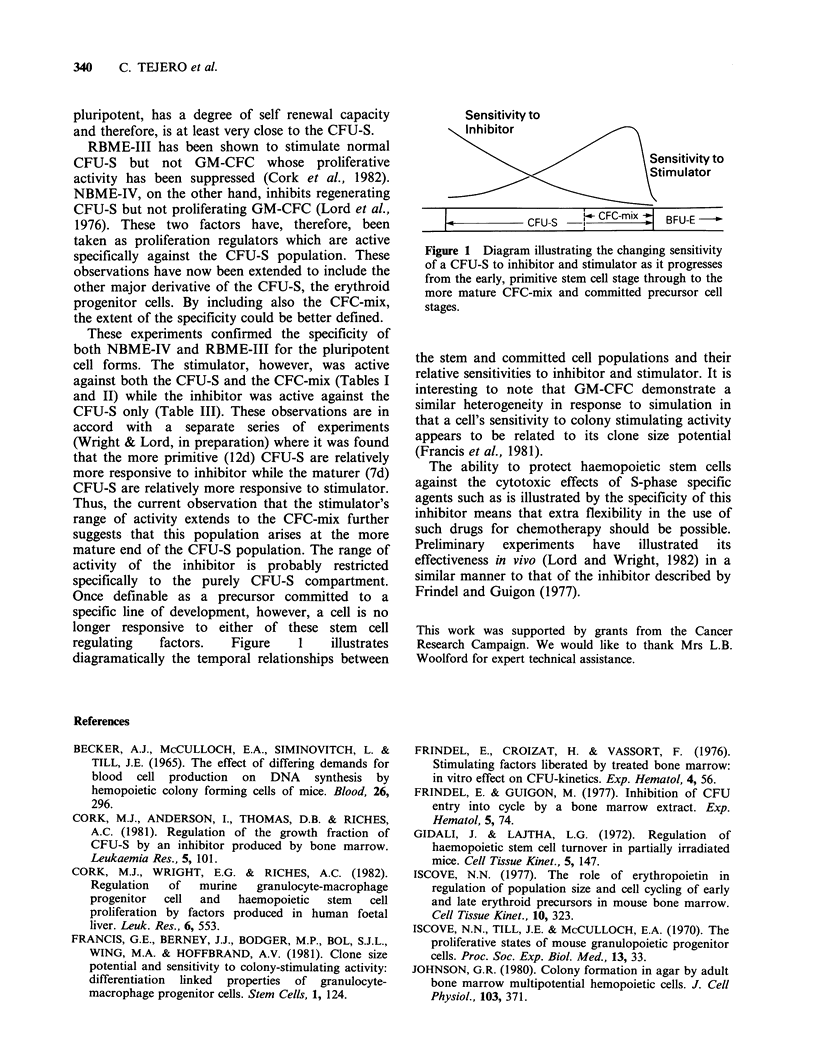

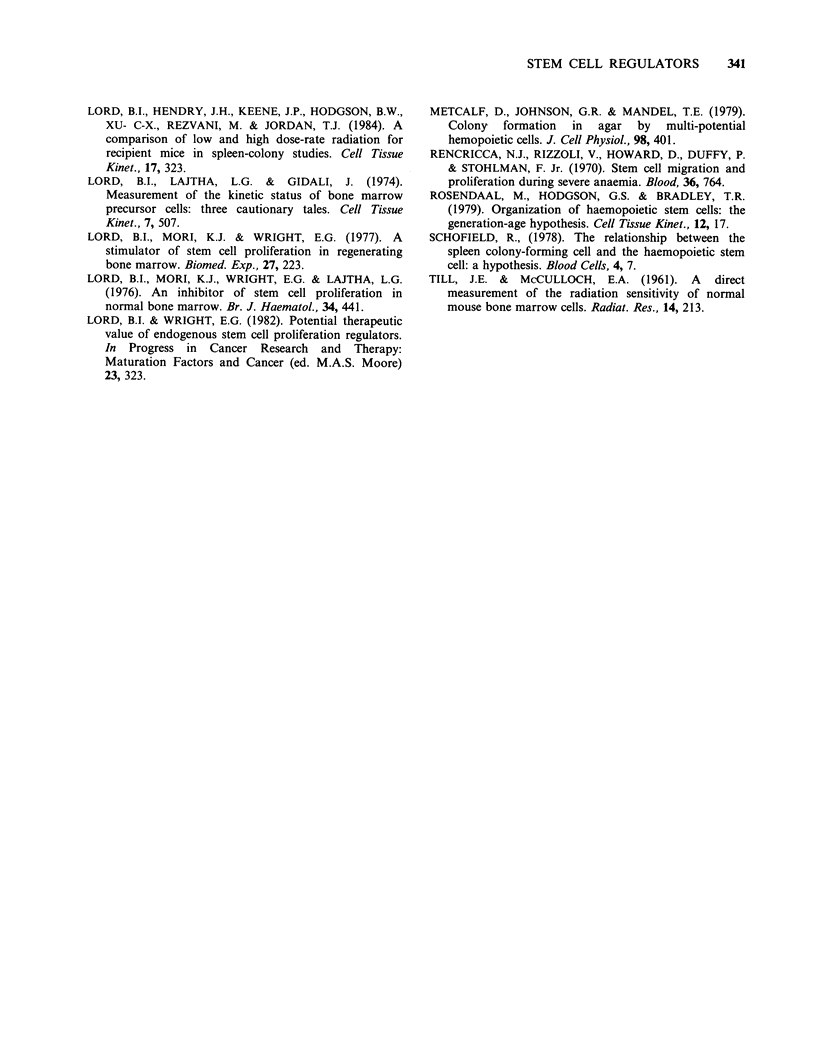


## References

[OCR_00582] BECKER A. J., MCCULLOCH E. A., SIMINOVITCH L., TILL J. E. (1965). THE EFFECT OF DIFFERING DEMANDS FOR BLOOD CELL PRODUCTION ON DNA SYNTHESIS BY HEMOPOIETIC COLONY-FORMING CELLS OF MICE.. Blood.

[OCR_00595] Cork M. J., Wright E. G., Riches A. C. (1982). Regulation of murine granulocyte-macrophage progenitor cell and haemopoietic stem cell proliferation by factors produced in human fetal liver.. Leuk Res.

[OCR_00589] Cork M., Anderson I., Thomas D. B., Riches A. (1981). Regulation of the growth fraction of CFU-S by an inhibitor produced by bone marrow.. Leuk Res.

[OCR_00602] Francis G. E., Berney J. J., Bodger M. P., Bol S. J., Wing M. A., Hoffbrand A. V. (1981). Clone size potential and sensitivity to colony-stimulating activity: differentiation-linked properties of granulocyte-macrophage progenitor cells.. Stem Cells.

[OCR_00609] Frindel E., Croizat H., Vassort F. (1976). Stimulating factors liberated by treated bone marrow: in vitro effect on CFU kinetics.. Exp Hematol.

[OCR_00614] Frindel E., Guigon M. (1977). Inhibition of CFU entry into cycle by a bone marrow extract.. Exp Hematol.

[OCR_00619] Gidali J., Lajtha L. G. (1972). Regulation of haemopoietic stem cell turnover in partially irradiated mice.. Cell Tissue Kinet.

[OCR_00624] Iscove N. N. (1977). The role of erythropoietin in regulation of population size and cell cycling of early and late erythroid precursors in mouse bone marrow.. Cell Tissue Kinet.

[OCR_00630] Iscove N. N., Till J. E., McCulloch E. A. (1970). The proliferative states of mouse granulopoietic progenitor cells.. Proc Soc Exp Biol Med.

[OCR_00635] Johnson G. R. (1980). Colony formation in agar by adult bone marrow multipotential hemopoietic cells.. J Cell Physiol.

[OCR_00642] Lord B. I., Hendry J. H., Keene J. P., Hodgson B. W., Xu C. X., Rezvani M., Jordan T. J. (1984). A comparison of low and high dose-rate radiation for recipient mice in spleen-colony studies.. Cell Tissue Kinet.

[OCR_00649] Lord B. I., Lajtha L. G., Gidali J. (1974). Measurement of the kinetic status of bone marrow precursor cells: three cautionary tales.. Cell Tissue Kinet.

[OCR_00655] Lord B. I., Mori K. J., Wright E. G. (1977). A stimulator of stem cell proliferation in regenerating bone marrow.. Biomedicine.

[OCR_00660] Lord B. I., Mori K. J., Wright E. G., Lajtha L. G. (1976). Inhibitor of stem cell proliferation in normal bone marrow.. Br J Haematol.

[OCR_00672] Metcalf D., Johnson G. R., Mandel T. E. (1979). Colony formation in agar by multipotential hemopoietic cells.. J Cell Physiol.

[OCR_00677] Rencricca N. J., Rizzoli V., Howard D., Duffy P., Stohlman F. (1970). Stem cell migration and proliferation during severe anemia.. Blood.

[OCR_00682] Rosendaal M., Hodgson G. S., Bradley T. R. (1979). Organization of haemopoietic stem cells: the generation-age hypothesis.. Cell Tissue Kinet.

[OCR_00692] TILL J. E., McCULLOCH E. A. (1961). A direct measurement of the radiation sensitivity of normal mouse bone marrow cells.. Radiat Res.

